# Use of handheld X-ray fluorescence as a non-invasive method to distinguish between Asian and African elephant tusks

**DOI:** 10.1038/srep24845

**Published:** 2016-04-21

**Authors:** Kittisak Buddhachat, Chatchote Thitaram, Janine L. Brown, Sarisa Klinhom, Pakkanut Bansiddhi, Kitichaya Penchart, Kanita Ouitavon, Khanittha Sriaksorn, Chalermpol Pa-in, Budsabong Kanchanasaka, Chaleamchat Somgird, Korakot Nganvongpanit

**Affiliations:** 1 Animal Bone and Joint Research Laboratory, Department of Veterinary Biosciences and Public Health, Faculty of Veterinary Medicine, Chiang Mai University, Chiang Mai 50100, Thailand; 2 Center of Excellence in Elephant Research and Education, Faculty of Veterinary Medicine, Chiang Mai University, Chiang Mai 50100, Thailand; 3 Maesa Elephant camp, 119/9, Tapae Road, Muang, Chiang Mai 50100, Thailand; 4 Smithsonian Conservation Biology Institute, Center for Species Survival, 1500 Remount Road, Front Royal, VA 22630, USA; 5 DNP Wildlife Forensic Science Unit (DNP-WIFOS), Wildlife Research Division, Wildlife Conservation Office, Department of National Parks, Wildlife and Plant Conservation. 61 Phaholyothin Rd., Chatuchak Bangkok 10900 Thailand; 6 Wildlife Research Division, Wildlife Conservation Office, Department of National Parks, Wildlife and Plant Conservation. 61 Phaholyothin Rd., Chatuchak Bangkok 10900 Thailand

## Abstract

We describe the use of handheld X-ray fluorescence, for elephant tusk species identification. Asian (n = 72) and African (n = 85) elephant tusks were scanned and we utilized the species differences in elemental composition to develop a functional model differentiating between species with high precision. Spatially, the majority of measured elements (n = 26) exhibited a homogeneous distribution in cross-section, but a more heterologous pattern in the longitudinal direction. Twenty-one of twenty four elements differed between Asian and African samples. Data were subjected to hierarchical cluster analysis followed by a stepwise discriminant analysis, which identified elements for the functional equation. The best equation consisted of ratios of Si, S, Cl, Ti, Mn, Ag, Sb and W, with Zr as the denominator. Next, Bayesian binary regression model analysis was conducted to predict the probability that a tusk would be of African origin. A cut-off value was established to improve discrimination. This Bayesian hybrid classification model was then validated by scanning an additional 30 Asian and 41 African tusks, which showed high accuracy (94%) and precision (95%) rates. We conclude that handheld XRF is an accurate, non-invasive method to discriminate origin of elephant tusks provides rapid results applicable to use in the field.

Elephants are keystone species that modify habitats by converting forests to grassland, create water holes in times of drought, and spread the seeds of plants. They also are umbrella species, as the conservation of elephants preserves not only habitat, but other species within. Last, they are flagship species and raise awareness for action and funding of conservation efforts. Despite their importance, wild elephant populations are being decimated by poaching for ivory^1^,^2^,^3^,^4^,^5^,^6^. In 1989, the Convention on International Trade in Endangered Species (CITES) prohibited the trade in ivory except samples from elephants that died naturally or came from captive animals. Nevertheless, the sale of illegal ivory is lucrative and a major threat to elephant survival, especially the African species (*Loxodonta africana*)^5^,^6^. In 2015, the African elephant was placed in Appendix I of CITES, except populations in Botswana, Namibia, South Africa and Zimbabwe, which are included in Appendix II because of their large population numbers. The African elephant also is on the wildlife protection list of Thailand Wildlife Preservation and Protection Act 2015. There is little doubt that today many African elephant populations are at serious risk. For example, in 2011, 25,000 African elephants were killed for ivory, and more than 30,000 in 2012. African forest elephants are particularly vulnerable; over 60% have been killed for the ivory trade in the last decade^4^. Asian elephants (*Elephas maximus*) are in Appendix I of CITES, in Schedule I of the Indian Wildlife (Protection) Act 1972, and on the wildlife protection list of the Thailand Wildlife Preservation and Protection Act of 1992. Although habitat destruction is the main threat to Asian elephant survival^6^, poaching of males for ivory also is a significant problem^6^. Controlling the illegal trade in ivory is essential, but difficult because some ivory sales are legal and there are few controls to prevent illegal ivory from being traded alongside legal stocks^1^,^2^. Thus, to combat the illegal ivory trade, it is crucial to know the source of tusks being sold.

Initially, measurement of Schreger lines and their subtended angles in cross-section was used to visually determine species of ivory sources, but it is not effective on pieces that have been sculpted or damaged^7^. In 2003, a method was developed to extract DNA from elephant ivory, which led to an ability to differentiate between Asian from African tusks, as well as the geographical location of where the ivory originated (at least for African elephants)^8^,^9^. Although today DNA analysis is considered the gold standard for tusk species identification, the technique requires destruction of a portion of the sample and the process of extraction is difficult, with failure of DNA to amplify being one technical problem^10^,^11^. In addition, this approach requires considerable technical expertise and equipment, and is time-consuming. Therefore, a simpler, non-destructive method for identifying the species of elephant tusks could aid forensic and law enforcement agencies in controlling the sale of illegal elephant ivory.

X-ray fluorescent (XRF) is an ideal technique for this purpose because it does not require chemical or mechanical destruction of samples, and offers results that are almost immediate. It is a method that simultaneously detects multiple elements in a sample, and already has been shown capable of discriminating between the teeth of Asian elephants and those of 15 other species^12^. Other studies have used a variety of elemental analysis techniques, including glancing incidence XRF, instrumental neutron activation analysis, particle induced X-ray emission, and stable isotope analysis, to distinguish between elephant tusks from varied geographical regions in Africa^13^,^14^,^15^,^16^. About a decade ago, Singh *et al*.^17^ used XRF to show that the elements strontium (Sr) and hafnium (Hf) were significantly higher in African than in Asian ivory (n = 5 samples each). And using principal component analysis of nine elements identified by XRF in Asian (n = 12), African (n = 6) and unknown (n = 1) tusks, Kautenburger^18^ identified two components that accounted for 84% of the variance in distinguishing species. Together, these results suggested it is feasible to use multi-element analyses to distinguish ivory samples from different elephant populations. However, prior work was based on relatively small sample sizes, so the power of XRF to identify tusk species origin has yet to be definitively established.

Our goal was to use a handheld XRF analyzer to conduct a multi-elemental analysis on a larger number of tusks (102 Asian, 126 African) to determine the capacity of this technique to distinguish between Asian and African elephant ivory. There are several advantages to using a handheld XRF analyzer: it is non-destructive, samples can be scanned *in situ* in the field, it does not require specialized laboratory equipment or expertise, and results are available almost immediately. Thus, handheld XRF may be a more practical and cost effective alternative to DNA analyses to determine origin of elephant ivory samples.

## Results

### Comparison of elemental composition between gender and tusk regions

**Figure 1:**
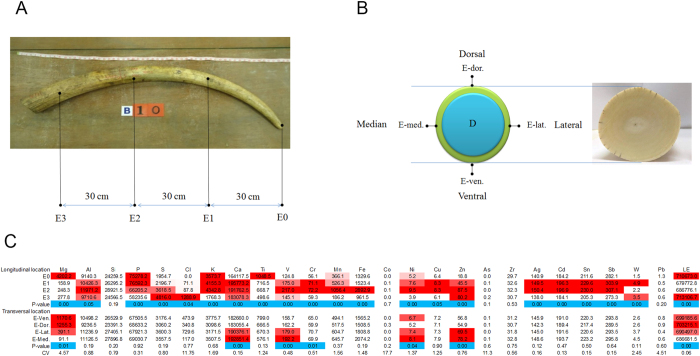
Schematics illustrate longitudinal (**A**) and transversal (**B**) XRF scanning sites of the enamel layer on elephant tusks for Asian and African elephants combined. Comparison of elemental levels (ppm) among longitudinal and transversal sites, and coefficients of variation (CV) are shown in (**C**). Significant *p* values (<0.05) are highlighted in blue, with concentration differences highlighted by the level of red intensity. (Mg = magnesium, Al = aluminum, Si = silicon, P = phosphorous, S = sulfur, Cl = chlorine, K = potassium, Ca = calcium, Ti = titanium, V = vanadium, Cr = chromium, Mn = manganese, Fe = iron, Co = cobalt, Ni = nickel, Cu = copper, Zn = zinc, As = arsenic, Zr = zirconium, Ag = silver, Cd = cadmium, Sn = tin, Sb = antimony, W = tungsten, Pb = lead, LE = light element).

**Figure 2:**
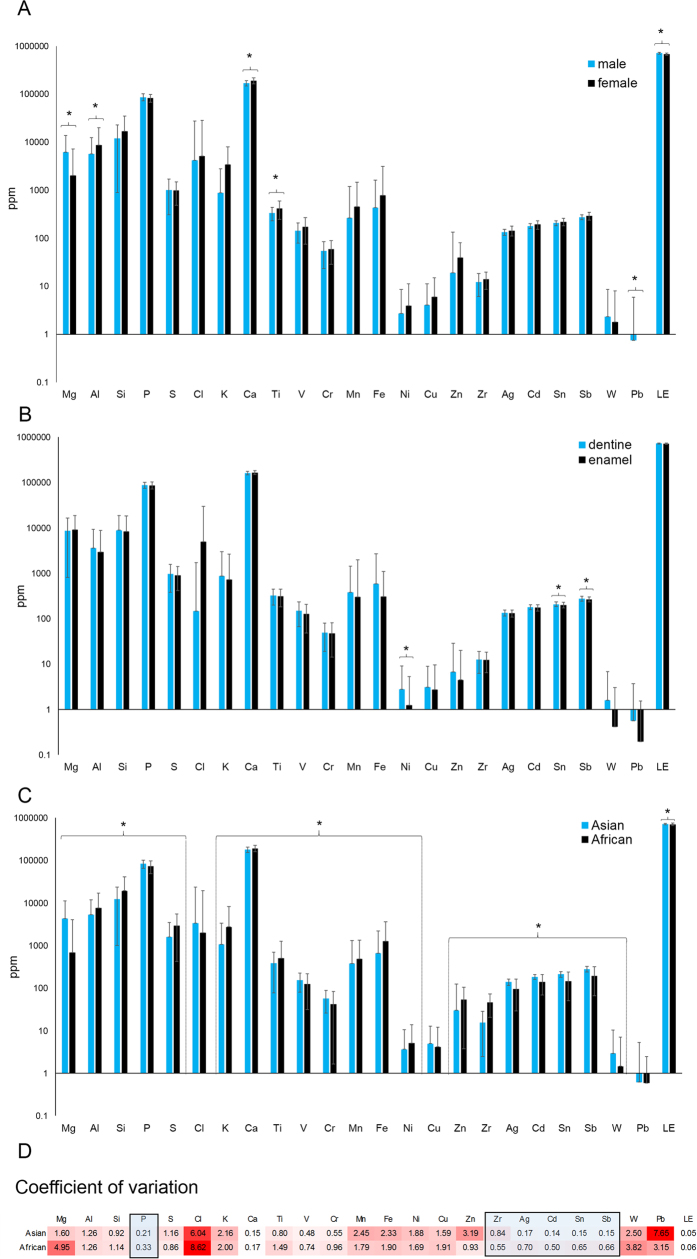
Differences in elemental composition between genders (Asian only, **A**), cross-sectional layers (**B**) and species (**C**). Coefficients of variation (CV), with darker red boxes indicating higher CVs. Elements in the blue boxes are those used as a denominator in creating the discriminant equation (**D**). (Mg = magnesium, Al = aluminum, Si = silicon, P = phosphorous, S = sulfur, Cl = chlorine, K = potassium, Ca = calcium, Ti = titanium, V = vanadium, Cr = chromium, Mn = manganese, Fe = iron, Ni = nickel, Cu = copper, Zn = zinc, Zr = zirconium, Ag = silver, Cd = cadmium, Sn = tin, Sb = antimony, W = tungsten, Pb = lead, LE = light element).

The locations for determining the distribution of elements in tusk samples are shown in Fig. 1A,B. Data in Fig. 1 were combined across species and gender. All but five elements; aluminum (Al), silicon (Si), cobalt (Co), arsenic (As), zirconium (Zr) and lead (Pb) differed in the longitudinal direction (E0–E3, Fig. 1C). A higher amount of sulfur (S), chlorine (Cl) and zinc (Zn) were observed at the base of the tusk, whereas magnesium (Mg), phosphorous (P) and titanium (Ti) concentrations were highest at the tip (p < 0.05). Other elements were more concentrated in the middle (E1 and/or E2) sections of the tusk (p < 0.05). In the transverse direction, calcium (Ca), vanadium (V), chromium (Cr), nickel (Ni) and Zn were highest along the medial plane, whereas Mg was highest in the ventral plane (p < 0.05). The ratio of Ca/P in the longitudinal direction was not different and ranged from 2.6–2.7. By contrast, the ratio of Ca/P in transversal location differed significantly with the highest ratio at the base (3.14) compared to the tip of tusk (2.18). A number of toxic heavy metals were detected in both Asian and African tusks: As, cadmium (Cd) and Pb. Cd displayed a preferential accumulation in E1 and E2 (p < 0.05), whereas As and Pb were non-significantly dispersed along the length of the tusk (p > 0.05). Between enamel and dentine layers, only Ni, tin (Sn) and antimony (Sb) were distributed unequally (Fig. 2B).

A gender comparison of Asian tusks found only five elements differed between male and female samples (E0–E3 data combined): a significantly larger proportion of Al, Ca and Ti in females, and a higher proportion of Mg and Pb in males (Fig. 2A).

**Figure 3:**
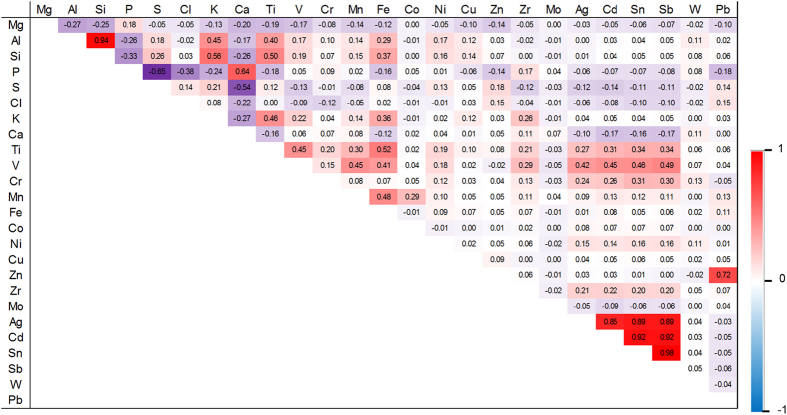
Pearson’s correlation coefficients between each element for Asian and African elephant tusks combined, as determined by XRF analysis. Colored boxes indicate direction and strength of each correlation. (Mg = magnesium, Al = aluminum, Si = silicon, P = phosphorous, S = sulfur, Cl = chlorine, K = potassium, Ca = calcium, Ti = titanium, V = vanadium, Cr = chromium, Mn = manganese, Fe = iron, Co = cobalt, Ni = nickel, Cu = copper, Zn = zinc, Zr = zirconium, Mo = molybdenum, Ag = silver, Cd = cadmium, Sn = tin, Sb = antimony, W = tungsten, Pb = lead).

A number of elements were significantly correlated when data for species and gender were combined (Fig. 3). The strongest positive correlations (R > 0.70) were observed between Al and Si, and between Pb and Zn. There also were a number of co-relationships among several of the heavy metals, silver (Ag), Cd, Sn and Sb. The highest negative correlations (R > −0.5) were observed between S with both Ca and P.

### Distinguishing between tusks from Asian and African elephants

**Figure 4:**
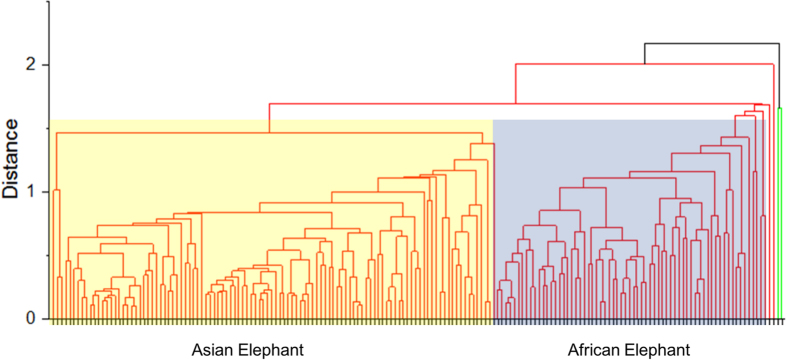
Hierarchical cluster analysis of individual elemental compositions obtained from XRF scanning of Asian and African tusks.

**Figure 5:**
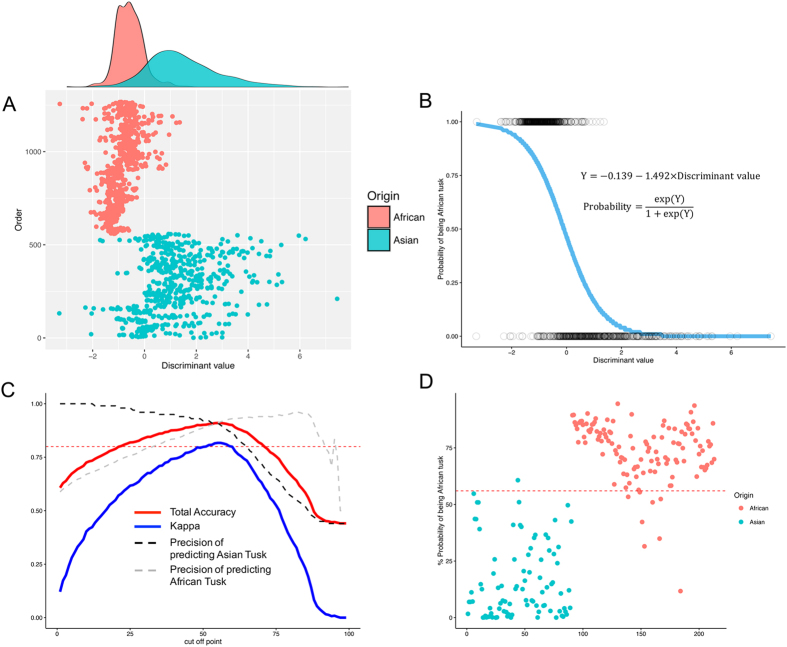
Effectiveness of the elemental composition data for estimating origin of elephant tusks. (**A**) Scatter plot and density of values using discriminant analysis with Zr as the denominator based on scans of 72 Asian and 85 African tusks. (**B**) Predicting Asian or African tusk origins (Asian = 0, African = 1) as a function of the discriminant values (DV) subjected to Bayesian logit regression, referred as to a Bayesian hybrid classification model. Data of DV are indicated by the dots according to tusk species. (**C**) The set of cut-off values for predicting Asian or African tusk origin. Dashed red line shows the level of kappa at 0.8. (**D**) Scatter plot testing the reliability of the model for estimating tusk origin for 30 Asian and 41 African tusks with a cut-off value of 55% (dashed red line).

Of the 24 elements measured, only three, copper (Cu), Cl, and Pb exhibited no difference between Asian and African tusks (Fig. 2C). Ca was higher in African tusks and P was higher in Asian tusks, leading to a higher ratio of Ca to P in African tusks (2.16 and 2.62, respectively). The first step towards determining the feasibility of element profiling for distinguishing between Asian and African tusks involved hierarchical cluster analysis (Fig. 4), which grouped data into ‘Asian’ and ‘African’ (Fig. 5A). Data for 157 tusks were subjected to discriminant analysis, and based on the criteria for selecting optimal denominators to create ratios, Zr was the most suitable (Table 1; Fig. 1C). Although the ‘Combined ratio’ gave the highest successive discrimination at 89.8%, its ‘Valid data’ for entering into the process of creating the function was only 79.4%, and it required 24 ratios for tusk prediction (Table 1). By contrast, 97.4% of the data were valid using Zr as the denominator and the equation required only nine elemental ratios with an 87.6% accuracy rate (Table 1). Altogether, the best equation for discriminating Asian from African elephant tusks was as follows:


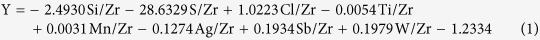


The equations for transforming the discriminant value to a probability of being of African origin through the Bayesian logit model are shown in Fig. 5B. The average probability was 0.24 and 0.75 for being Asian or African tusks, respectively. The Asian tusks exhibited a higher degree of variation in both discriminant values and probability, which was evidenced by the overlapping areas in Fig. 5A, and the continuous lines of probability in Fig. 5B. Thus, a cut-off value of 55% probability of being African tusk was used as boundary line for sorting Asian and African because it provided high kappa statistic (0.82) concomitant to high accuracy and average agreement rates (Fig. 5C). A predicted probability of less than 55% classified tusks as ‘Asian’, whereas those more than 55% were predictive of ‘African’ tusks. This model using the cut-off value correctly categorized Asian and African tusks at 89% and 93%, respectively (91% total accuracy rate) which this model was referred as to “Bayesian hybrid classification model”. To validate the model, an additional 30 Asian and 41 African elephant tusks were scanned and the values subjected to Bayesian hybrid classification model as an independent study, which resulted in similar high accuracy and low error rates (Table 2, Fig. 5D).

## Discussion

Identifying whether confiscated tusks are of Asian or African origin is crucial to stemming the illegal ivory trade, and today generally relies on DNA analyses. We provide evidence that elemental analysis using XRF can serve as an alternative diagnostic tool for tusk species identification, which may have more utility on a larger scale because it does not rely on sophisticated laboratory equipment or expertise, and can be done inexpensively under field conditions. An XRF scan takes less than 5 minutes, and usually involves three scans along the length of the tusk. The data are then entered into a computer program that predicts tusk origin. A total of 72 Asian and 85 African tusks were used to create the model, which was then validated by scanning an additional 30 Asian and 41 African tusks, resulting in high accuracy (94%) and precision (95%) rates. Altogether, we predict this technique will have tremendous application as a practical alternative to DNA analyses for tusk identification, allowing for better monitoring of the illegal ivory trade.

To develop this technique, we scanned the distribution of each element in whole tusks of both species. Only seven elements (Mg, Ca, V, Cr, Ni, Zn and LE) were significantly different in cross-section, whereas 21 of 26 elements scanned longitudinally had a heterogeneous distribution. Of the elements used in the discriminant equation, several varied in the longitudinal (Mg, S, Ti, V, Sb, W, LE) and transverse (Mg, V, LE) directions. Thus, tusk growth zones appear to incorporate elements at different rates so scanning site could affect results. In fact, Kautenburger *et al*.(13) recommended scanning the same area of a tusk (e.g., the tooth neck). However, we obtained excellent results by combining data from several scan sites along the tusk length, from tip to base, ignoring dorsal, ventral, medial and lateral planes as the elemental distribution there was homogenous. In this study, whole tusks were scanned, so discriminant analyses were based on the elemental composition of enamel only. Whether this approach will work for carved ivory remains to be determined; however, we believe it should because most elements exhibited a homogeneous distribution in cross-section. The few elements that differed between dentine and enamel (Ni, Sb, Sn) all were significantly different between Asian and African elephant tusks, so are not expected to affect discrimination success.

In addition to scan site, we were interested in potential gender effects on elemental composition. Both male and female African elephants grow tusks, whereas in Asian elephants, only males have tusks; females have small tusks, called tushes. We only had tusk material of known gender from Asian elephants, which revealed that Mg, Al, Ca, Ti and Pb were sexually dimorphic. Magnesium is integral to cellular and systemic mammal physiology, and stored within bones (50%) and soft tissues (47%) (8). It is involved in ATP metabolism and as a cofactor for over 300 enzymes. In our study, a larger proportion of Mg was observed in male than female samples, which could be related to: (i) male tusks having a higher growth rate and a larger size than female tusks, thus Mg in males may be more widely distributed when compared to that of females; or (ii) because bone (teeth as well) is a predominate storage tissue for Mg in the body, and males have higher energy needs than females, males may require a higher Mg storage than females (8). A recent study suggested Mg in teeth affects the quality and structure of hard dental tissues, particularly the enamel layer (9), although the mechanism is unclear (8). The proportion of Ca was higher in female than male tusks, while P did not differ significantly, resulting in a higher Ca/P ratio in female tusks. In addition to a gender difference, we found the Ca/P ratio was highest at the base and decreased towards the tip of tusk, which might be important in providing strength at the base for protecting the breaking of tusk from digging, fighting and protecting the trunk. Whether female tusks are stronger than those of males because of this elemental difference remains to be determined. Lead levels in male tusks also were significantly higher than in female samples, which agrees with previous studies of sex differences in Pb in human bone^19^,^20^. Also higher in female tusks were Al and Ti, two elements that can result from environmental contamination^21^ and thus warrant further study. However, a sex bias for the other elements was not observed.

The finding of heavy metals, Pb and As, in tusks suggests another type of environmental contamination. Main sources of Pb come from lead-based fuels, spent ammunition, and wastewater^22^,^23^. Interestingly, there was a strong positive correlation between Pb and Zn (R = 0.72), suggesting they bio-accumulate. According to Pemmer *et al*.^24^, high levels of Zn and Pb are found in the cement line of the mineralized bone matrix in humans, and are believed to be due to accumulation by two ways: (i) direct incorporation into hydroxyapatite crystals; and (ii) binding to proteins with a high affinity to these elements. For example, increased Pb concentrations in the cement lines could be due to the osteocalcin, which has a higher affinity to Pb than to Ca, even at low Pb levels^25^,^26^, while Zn is part/cofactor of enzymes like matrix metalloproteinases (MMPs) that are mainly responsible for degradation of collagen during the remodeling cycle of bone^27^ and bone alkaline phosphatase^28^.

When comparing the elemental profiles between Asian and African elephant tusks by student’s t-test, a number of differences were observed, and in fact only Cl, Cu and Pb were the same concentration. A previous study using XRF found elemental differences between African elephant tusk samples from Namibia and Zimbabwe, and two samples of Asian elephants from northern Thailand, although exact elemental differences were not described^18^. Another study reported Sr and Hf concentrations were higher in African than in Asian ivory^17^. Unfortunately, neither Sr or Hf were detected by the handheld XRF unit used in this study because the operating voltage of the handheld unit is 15 and 40 kV and a higher voltage (50 kV) is needed to detect Sr^12^. Hf was not detected because our unit lacked the needed sensitively; Sr is not detectible at less than 10 ppm. No doubt these species differences in elemental proportions are due to the varied geographic regions of Asia versus Africa, and elemental differences in water and food sources associated with regional types of soil, climate and plant species^19^,^21^,^29^,^30^. Vegetation in particular is a main source of elements in the diet, so differences in organic (amino acid) and inorganic substrates (elements including Ca, P, Mg, F, Co and Zn) could account for differential uptake in tusks^13^.

What the species differences allowed us to do was create an effective model for differentiating between Asian and African tusks. Data from all scans of 72 Asian and 85 African tusks were analyzed by a stepwise discriminant analysis, and the discriminant values subjected to Bayesian binary regression to predict the probability that a tusk would be of Asian or African origin. This Bayesian hybrid classification model had a high accuracy rate (94.4%) with low error (95.5% precision rate) when used with a cut-off of 55%. Successful discrimination using Zr as the denominator for all ratios was due to its homogeneous distribution throughout whole tusk, a low inter-individual variation for each species, and the sizeable difference between Asian and African tusks in total concentrations. Use of Zr as the denominator also resulted in the best combination of a high eigenvalue, high successive discrimination value and the least number of ratios needed for discrimination. In this paper, Bayesian binary regression model was allowed to predict probability of being African or Asian and increase the reliability of the origin estimation of tusk.

In conclusion, species identification of elephant tusks has long been a problem for law enforcement authorities. Here we describe a proof of concept study to determine the feasibility of using handheld XRF for the determination of tusk species. Next steps will be to test the discriminatory ability of XRF to identify the origin of carved/cut ivory, which based on our results appears likely.

## Limitations

The handheld XRF equipment is convenient to transport and use in the field; however, it only scans surface elements at a ~2 cm depth of enamel by 40 kV of X-ray energy. Thus, if the surface is treated with chemical agents or protectants that contain elements detected by XRF, the percentages could be altered and lead to inaccurate measurements and false identification. For this reason, all samples in this study were untreated and cleaned of any dirt and debris before scanning. Another method is to use the elemental concentration based on known scanning depths for each element^31^. The probing volumes in tusks would vary for elements with strongly different atomic numbers, so knowing actual concentrations would avoid alterations in values from additive chemicals because it is a direct and not a relative quantification. However, the handheld XRF machine does not provide probing volumes so we could not calculate elemental concentrations. Rather, we used hierarchical cluster analysis followed by stepwise discriminant analysis and Bayesian logit model with a cut-off value to create a functional algorithm that was highly accurate, but did not rely on concentration determination. However, because of the heterogeneous distribution of some elements along tusk locations, and slight deviations in probing depth per scan, we suggest scanning several points along the tusk for average value to reduce the variation. We also recommend that for either method, one should be confirming that major elements, like Ca and P and their ratios are within normal ranges before accepting predictions. And whenever possible, other methods, such as morphological evaluations or molecular tools should be used for unclear samples. Last, although not a limitation per se, it is clear that the light element matrix of the tusks not quantifiable by XRF analysis has an impact on some elemental proportions, so use of methodology to quantify these components might provide additional information that could lead to further improvements in discrimination functions.

## Methods

### X-ray fluorescence measurement

**Figure 6:**
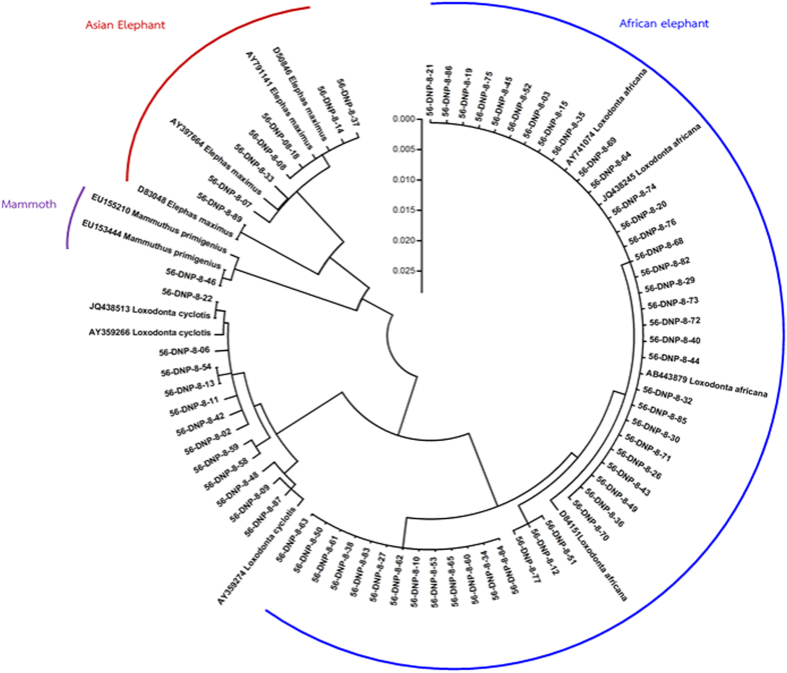
Phylogenetic tree based on cytochrome b sequence.

Elemental analyses were conducted using a handheld XRF analyzer (DELTA Premium, Olympus, USA) with a silicon drift detector that measures elements from magnesium (12 Mg) through bismuth (83 Bi) on the periodic table. The collimator size was set at 0.3 mm for analysis-area diameter, and operating voltages of 15 and 40 kV were used as the source of incident radiation. These voltages are insufficient to discriminate elements with an atomic number less than 12, which we refer to as light elements (LE). The data set of each element derived from XRF was reported as relative, semi-quantified values, given as a percentage of the total, which were then converted to ppm (% × 10,000). A total of 228 tusks were used in this study, from 102 Asian (78 males, 9 females, 15 unknown sex) and 126 African (unknown sex) elephants. Eighty-eight Asian elephant tusks were obtained from the Elephant Research and Education Center, Faculty of Veterinary Medicine, Chiang Mai University, Chiang Mai, Thailand. Fourteen Asian and 126 African elephant tusks were obtained from the Wildlife Research Division, Wildlife Conservation Office, Department of National Parks, Wildlife and Plant Conservation, Bangkok, Thailand. African elephant tusks were confirmed by DNA analyses using cytochrome b sequencing (Fig. 6)^31^, while Asian elephant tusks were of known origin from locations in Thailand.

To determine the homogeneity of elements at locations along the tusk, elemental distributions based on scanning location were assessed in 30 Asian (male, female) and 15 African (male) elephant tusks. For this analysis, tusks were divided into four regions: the tip of tusk was the first point (E0), with subsequent points at 30 cm-length distances (E1, E2 and E3) (Fig. 1A). Because tusks are curve-shaped, both sides at each location were scanned in the dorsal (E-Dor), ventral (E-Ven), medial (E-Med) and lateral (E-Lat) planes (Fig. 1B). To examine differences between sex and also between enamel and dentin layers in elemental composition, 87 Asian elephant tusks (78 males, 9 females) were scanned at each site in both longitudinal and transverse directions. At least three sites at each location were measured for elemental composition, resulting in a total of 1,512 scans. Elemental data are presented as ppm.

The methods were carried out in accordance with the approved guidelines. The experimental protocol was approved (2014) by the Scientific and Ethics Committee, Center of Excellence in Elephant Research and Education, Faculty of Veterinary Medicine, Chiang Mai University, Chiang Mai Thailand.

### Statistical analyses

#### Determination of difference in elements in tusk

Differences in mineral composition in tusks based on gender (males versus females), species (Asian versus African) and location (enamel versus dentin) were analyzed using t-tests. For the effect of scanning location on tusk elemental profiles, data sets were analyzed using one-way ANOVA. A p-value of <0.05 was considered statistically significant.

#### Establishment of the hybrid classification model

To create an effective equation for differentiating Asian and African tusks, data obtained from scanning 157 tusks (72 Asian and 85 African) were subjected to hierarchical cluster analysis and stepwise discriminant analysis^32^,^33^,^34^. Prior to creating the effective function, optimal ratios for use in the equations were identified by the following criteria: (i) the element was detected in tusks of both Asian and African with a coefficient of variation (CV) less than 1; (ii) its distribution was homogenous or slightly heterogeneous in every dimension, including longitudinal directions, transversal locations and cross-section (dentine and enamel); and (iii) the element needed to be significantly different between Asian and African tusks. As a result, Zr, P, Ag, Cd, Sn and Sb were found to be suitable ratio denominators. The accuracy rates of each equation were tested by “leave one out classification” to evaluate the capacity of each equation to discriminate Asian and African tusk^35^,^36^,^37^. Subsequently, the discriminant values were subjected to Bayesian binary regression^38^,^39^,^40^ to determine the probability of being of Asian or African origin. The independent variables (y_i_) followed a Bernoulli distribution. In this study, the independent variables of Asian and African tusk were designated as 0 and 1, respectively. The model is as follows:


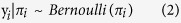


where *π* is defined as:


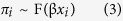


with the function F as an arbitrary cumulative distribution function (CDF), and β as the vector of unknown parameter of the regression model. The uncertainty of β is characterized through a probability distribution. The most useful specifications of F included the logistic, the normal (probit) and the extreme (complementary log log; cloglog) values as follows:


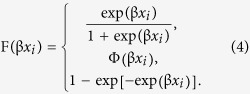


The link function defined the linear predictor as:


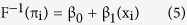


These three models after transformation as link functions are respectively: log(*π*_*i*_/(1 − *π*_*i*_), Φ^−1^(*π*_*i*_) and log(−log(1 − *π*_*i*_)). Link functions of logit, probit and cloglog were used for the probability of being ivory. In order to select the proper model for that propose, the Deviance Information Criterion (DIC) was used^41^. Firstly, a regression method was employed. Uncertainty about the value of β_0_, β_1_ in the modeling was estimated using an independent normal distribution with mean (μ) equal to zero and a high variance (*σ*^2^) (precision = 1/*σ*^2^ as 0.001) (to let the parameters vary in a large range) due to weakly informative prior distribution^42^:


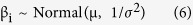


The estimation of the posterior distribution of parameters based on reasonable prior assumptions by Bayesian binary regression was conducted by Monte Carlo Markov Chain (MCMC) simulation^43^. The MCMC output of all the parameters passed the convergence test of Gelman and Rubin Diagnostic and Geweke Diagnostic, available for free with the CODA package implemented in R program^44^. We found that the logit showed the fittest model because of the lowest DIC. In this study, logit was allowed to predict the probability of being an African tusk.

#### Establishing the cut-off for classifying Asian and African tusks

The Bayesian hybrid classification model was established by discriminant analysis followed by Bayesian logit regression model for predicting the probability of a sample being an African tusk. However, some points around 50% probability could not be easily discriminated. So, a cut-off value was required to predict a tusk as being Asian or African. In this study, we used the kappa statistic^45^ for choosing the cut-off point, which showed a high reliability (kappa > 0.8). The accuracy and precision rates were calculated as follows:


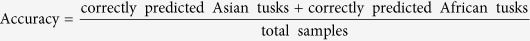



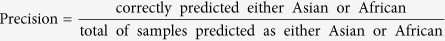


Finally, the criterion for selecting the optimal cut-off consisted of a (i) kappa statistic more than 0.8 and (ii) the highest accuracy and precision rates.

#### Evaluation of reliability of hybrid classification model

To validate the effectiveness of the Bayesian hybrid classification model, we scanned an additional 30 Asian and 41 African tusks. These tusks were scanned at three or more random points along an individual tusk. Subsequently, the averages of the independent set of data were predicted as to whether they were of Asian or African origin using the Bayesian hybrid classification model. The accuracy and precision were calculated.

## Additional Information

**How to cite this article**: Buddhachat, K. *et al*. Use of handheld X-ray fluorescence as a non-invasive method to distinguish between Asian and African elephant tusks. *Sci. Rep.* **6**, 24845; doi: 10.1038/srep24845 (2016).

## Figures and Tables

**Table 1:** Characterization of each discriminant function using the different elemental denominations.

Ratio of each element	Valid data^1^ (%)	Eigen value	Number of ratios used	Successive discrimination (%)
No ratio	100.0	1.297	11	87.5
To Zr	97.4	1.045	8	87.6
To P	100.0	0.618	12	84.9
To Ag	82.0	0.914	12	85.9
To Cd	93.7	0.809	13	85.5
To Sn	87.8	0.725	14	84.1
To Sb	87.8	0.684	12	83.8
Combined^2^	79.4	1.526	24	89.8

(P = phosphorous, Zr = zirconium, Ag = silver, Cd = cadmium, Sn = tin, Sb = antimony).

^1^Accepted data for entering into discriminant analysis.

^2^Ratios of Zr, P, Ag, Cd, Sn and Sb were pooled before analyzing through discriminant analysis.

**Table 2:** The classification results of assigning tusk origin through the hybrid classification model with cut off at 55% for samples used to create the discriminate equation and a separate set of independent samples used to validate the technique.

	Average probability (SD)	Prediction		Accuracy	Precision
		Asian	African		
Dependent study					
Asian (n = 72)	0.24 (0.23)	64 (89%)	8 (11%)		
African (n = 85)	0.75 (0.14)	6 (7%)	79 (93%)	91.08	91.12
Independent study					
Asian (n = 30)	0.16 (0.17)	29 (97%)	1 (3%)		
African (n = 41)	0.74 (0.13)	3 (7%)	38 (93%)	94.37	95.49

SD = standard deviation.

## References

[b1] Wittemyer G. . Illegal killing for ivory drives global decline in African elephants. Proc Natl Acad Sci USA 111, 13117–13121 (2014). 25136107 10.1073/pnas.1403984111 PMC4246956

[b2] Underwood F. M., Burn R. W. & Milliken T. Dissecting the illegal ivory trade: an analysis of ivory seizures data. Plos One. 8, e76539 (2013). 24250744 10.1371/journal.pone.0076539 PMC3799824

[b3] Burn R. W., Underwood F. M. & Blanc J. Global trends and factors associated with the illegal killing of elephants: A hierarchical bayesian analysis of carcass encounter data. Plos One. 6, e24165 (2011). 21912670 10.1371/journal.pone.0024165 PMC3166301

[b4] Maisels F. . Devastating decline of forest elephants in Central Africa. Plos One. 8, e59469 (2013). 23469289 10.1371/journal.pone.0059469 PMC3587600

[b5] Lemieux A. M. & Clarke R. V. The international ban on ivory sales and its effects on elephant poaching in Africa. Br J Criminol 49, 451–471 (2009).

[b6] Stiles D. The ivory trade and elephant conservation. Environmental Conservation 31, 309–321 (2004).

[b7] Espinoza E. O. & Mann M. J. The history and significance of the Schreger pattern in proboscidean ivory characterization. JAIC 32, 241–248 (1993).

[b8] Wasser S. K. . Assigning African elephant DNA to geographic region of origin: applications to the ivory trade. PNAS 101, 14847–14852 (2004). 15459317 10.1073/pnas.0403170101 PMC522003

[b9] Wasser S. K. . Using DNA to track the origin of the largest ivory seizure since the 1989 trade ban. PNAS 104, 4228–4233 (2007). 17360505 10.1073/pnas.0609714104 PMC1805457

[b10] Yang D. Y. & Watt K. Contamination controls when preparing archaeological remains for ancient DNA analysis. J Archaeol Sci 32, 331–336 (2005).

[b11] Toledano T. . An assessment of DNA contamination risks in New York City Medical Examiner facilities. J Forensic Sci 42, 721–724 (1997). 9243840

[b12] Nganvongpanit K., Brown J. L., Buddhachat K., Somgird C. & Thitaram C. Elemental analysis of Asian elephant (*Elephas maximus*) teeth using X-ray fluorescence and a comparison to other species *Biological Trace Element Research* Imprinting, 10.1007/s12011-12015-10445-x (2015). 26194819

[b13] Raubenheimera E. J. . Geographic variations in the composition of ivory of: the African elephant (*Loxodonta africana*). Arch Oral Biol 43, 641–647 (1998). 9758047 10.1016/s0003-9969(98)00051-x

[b14] Prozesky V. M. . Trace element concentration and distribution in ivory. Nuclear Instruments and Methods in Physics Research 104, 638–644 (1995).

[b15] Shimoyama M., Nakanishi T., Hamanaga Y., Ninomiya T. & Ozaki Y. Non-destructive discrimination between elephant ivory products and mammoth tusk products by glancing incidence X-ray fluorescence spectroscopy. J. Trace Microprobe Tech 16, 175–182 (1998).

[b16] Takeuchi T., Nakano Y. & Koike H. Neutron activation analysis of ivory of African elephants. J Radioanal Nucl Chem 235, 273–278 (1998).

[b17] Singh R. R., Goyal S. P., Khanna P. P., Mukherjee P. K. & Sukumar R. Using morphometric and analytical techniques to characterize elephant ivory. Forensic Sci Int 162, 144–151 (2006). 16891073 10.1016/j.forsciint.2006.06.028

[b18] Kautemburger R., Wannemacher J. & Muller P. Multi element analysis by X-ray fluorescence: A powerfull tool of ivory identification from various origins. J Radio Nuc Chem 260, 399–404 (2004).

[b19] Komarnicki G. J. Tissue, sex and age specific accumulation of heavy metals (Zn, Cu, Pb, Cd) by populations of the mole (*Talpa europaea* L.) in a central urban area. Chemosphere 41, 1593–1602 (2000). 11057686 10.1016/s0045-6535(00)00018-7

[b20] Massányi P., Tataruch F., Slameka J., Toman R. & Jurík R. Accumulation of lead, cadmium, and mercury in liver and kidney of the brown hare (Lepus europaeus) in relation to the season, age, and sex in the West Slovakian Lowland. J Environ Sci Health A Tox Hazard Subst Environ Eng 38, 1299–1309 (2003). 12916853 10.1081/ese-120021127

[b21] Mayzel B., Aizenberg J. & Ilan M. The elemental composition of demospongiae from the Red Sea, Gulf of Aqaba. Plos One. 9, e95775 (2014). 24759635 10.1371/journal.pone.0095775 PMC3997428

[b22] Parkpian P., Leong S. T., Laortanakul P. & Thunthaisong N. Regional monitoring of lead and cadmium contamination in a tropical grazing land site, Thailand. Environ Monit Assess 85, 157–173 (2003). 12828350 10.1023/a:1023638012736

[b23] Chanpiwat P. & Sthiannopkao S. Status of metal levels and their potential sources of contamination in Southeast Asian rivers. Environ Sci Pollut Res Int. 21, 220–233 (2014). 23807555 10.1007/s11356-013-1858-8

[b24] Pemmer B. . Spatial distribution of the trace elements zinc, strontium and lead in human bone tissue. Bone 57, 184–193 (2013). 23932972 10.1016/j.bone.2013.07.038 PMC3807669

[b25] Dowd T. L., Rosen J. F., Mints L. & Gundberg C. M. The effect of Pb 2+ on the structure and hydroxyapatite binding properties of osteocalcin. Biochim Biophys Acta 1535, 153–163 (2001). 11342004 10.1016/s0925-4439(00)00094-6

[b26] Dowd T. L., Rosen J. F., Gundberg C. M. & Gupta R. K. The displacement of calcium from osteocalcin at submicromolar concentrations of free lead. Biochim Biophys Acta 1226, 131–137 (1994). 8204659 10.1016/0925-4439(94)90020-5

[b27] Krane S. M. & Inada M. Matrix metalloproteinases and bone. Bone 43, 7–18 (2008). 18486584 10.1016/j.bone.2008.03.020

[b28] Yamaguchi M. Role of zinc in bone formation and bone resorption. J Trace Elem Exp Med 11, 119–135 (1998).

[b29] Brown C. J. . Environmental influences on the trace element content of teeth–implications for disease and nutritional status. Arch Oral Biol 49, 705–717 (2004). 15275858 10.1016/j.archoralbio.2004.04.008

[b30] Chiarenzelli J. . Multi-element and rare earth element composition of lichens, mosses, and vascular plants from the Central Barrenlands, Nunavut, Canada. Applied Geochemistry 16, 245–270 (2001).

[b31] Mantouvalou, I., Malzer, W. & Kanngieβer, B. Quantification for 3D micro X-ray fluorescence. *Spectrochim Acta B* **77**, 9–18 (2012).

[b32] Lee J. C. . Ivory identification by DNA profiling of cytochrome b gene. Int J Legal Med 123, 117–121 (2009). 18612647 10.1007/s00414-008-0264-0

[b33] Martiniaková M., Grosskopf B., Omelka R., Vondráková M. & Bauerová M. Differences among species in compact bone tissue microstructure of mammalian skeleton: use of a discriminant function analysis for species identification. J Forensic Sci 51, 1235–1239 (2006). 17199608 10.1111/j.1556-4029.2006.00260.x

[b34] Martiniaková M., Grosskopf B., Vondráková M., Omelka R. & Fabis M. Differences in femoral compact bone tissue microscopic structure between adult cows (*Bos taurus*) and pigs (*Sus scrofa* domestica). Anat Histol Embryol 35, 167–170 (2006). 16677211 10.1111/j.1439-0264.2005.00652.x

[b35] Moreno J. M., Roa H. I., Zavando D. & Galdames I. S. Determination of the species from skeletal remains through histomorphometric evaluation and discriminant analysis. Int J Morphol 30, 1035–1041 (2012).

[b36] Ogawa Y., Imaizumi K., Miyasaka S. & Yoshino M. Discriminant functions for sex estimation of modern Japanese skulls. J Forensic Leg Med 4, 234–238 (2013). 23622466 10.1016/j.jflm.2012.09.023

[b37] Lee U. Y., Kim I. B. & Kwak D. S. Sex determination using discriminant analysis of upper and lower extremity bones: New approach using the volume and surface area of digital model. Forensic Sci Int 253, 1–4 (2015). 26117502 10.1016/j.forsciint.2015.05.017

[b38] Alunni V., du Jardin P., Nogueira L., Buchet L. & Quatrehomme G. Comparing discriminant analysis and neural network for the determination of sex using femur head measurements. Forensic Sci Int 253, 81–87 (2015). 26093772 10.1016/j.forsciint.2015.05.023

[b39] Albert J. H. & Chib S. Bayesian analysis of binary and polychotomous response data. J Am Stat Assoc 88, 669–679 (1993).

[b40] Calle-Alonso F., Pérez C. J., Arias-Nicolás J. P. & Martín J. Computer-aided diagnosis system: A Bayesian hybrid classification method. Comput Methods Programs Biomed 112, 104–113 (2013). 23932384 10.1016/j.cmpb.2013.05.029

[b41] Dendukuri N. & Joseph L. Bayesian approaches to modeling the conditional dependence between multiple diagnostic tests. Biometrics 57, 158–167 (2001). 11252592 10.1111/j.0006-341x.2001.00158.x

[b42] Spiegelhalter D. J., Best N. G., Carlin B. P. & Van Der Linde A. Bayesian measures of model complexity and fit. J R Stat Soc Series B 64, 583–639 (2002).

[b43] Zellner A. & Rossi P. E. Bayesian analysis of dichotomous quantal response models. J Econometrics 25, 365–393 (1984).

[b44] Raftery A. E. & Lewis S. M. In Markov chain Monte Carlo in practice (eds Gilks W. R., Spiegelhalter D. J. & Richardson S.) 115–130 (Chapman and Hall, London, 1996).

[b45] Gelman A. & Shirley K. In Handbook of Markov chain Monte Carlo (eds Brooks S., Gelman A., Jones G. L., & X Meng L.) 163–174. (Chapman & Hall/CRC, 2011).

